# Correction: Fernández Blanco et al. A Photonic Immunosensor Detection Method for Viable and Non-Viable *E. coli* in Water Samples. *Microorganisms* 2024, *12*, 1328

**DOI:** 10.3390/microorganisms13091984

**Published:** 2025-08-26

**Authors:** Ana Fernández Blanco, Yolanda Moreno, Jorge García-Hernández, Manuel Hernández

**Affiliations:** 1 Lumensia Sensors S.L., 46020 Valencia, Spain; afernandez@lumensia.com; 2 Institute of Water and Environmental Engineering, Universitat Politècnica de València, 46022 Valencia, Spain; 3 Advanced Center for Food Microbiology, Biotechnology Department, Universitat Politècnica de València, 46022 Valencia, Spain; jorgarhe@btc.upv.es (J.G.-H.); mhernand@btc.upv.es (M.H.)

In the original publication [1], Figure 2 has been replaced by a new reference [53]. The citation [53] has now been inserted in paragraphs 4, 6 and 7 of Section 2.5. The rest of the figures and references have been renumbered accordingly.

The newly added reference [53] is as follows:

Fernández Blanco, A.; Hernández Pérez, M.; Moreno Trigos, Y.; García-Hernández, J. Specific and Simultaneous Detection of *E. coli* O157:H7 and Shiga-like Toxins Using a Label-Free Photonic Immunosensor. *Photonics* **2024**, *11*, 374.

There was a mistake in the original Figure 4A,B as published. The corrected Figure 4A,B (now Figure 3A,B) appear below.

The authors state that the scientific conclusions are unaffected. This correction was approved by the Academic Editor. The original publication has also been updated.

## Figures and Tables

**Figure 3:**
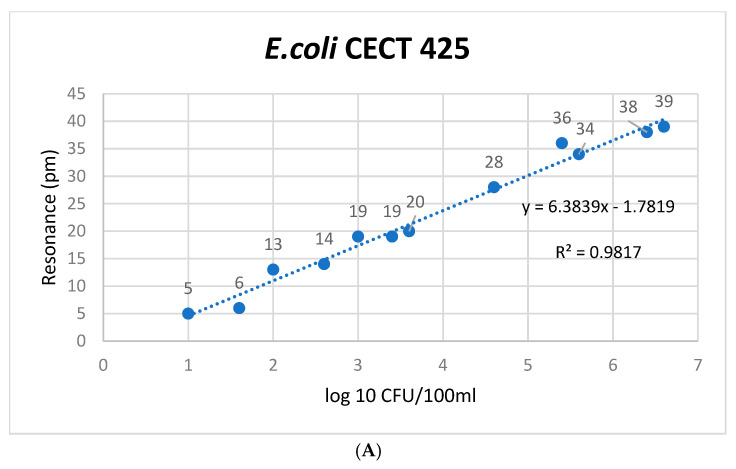

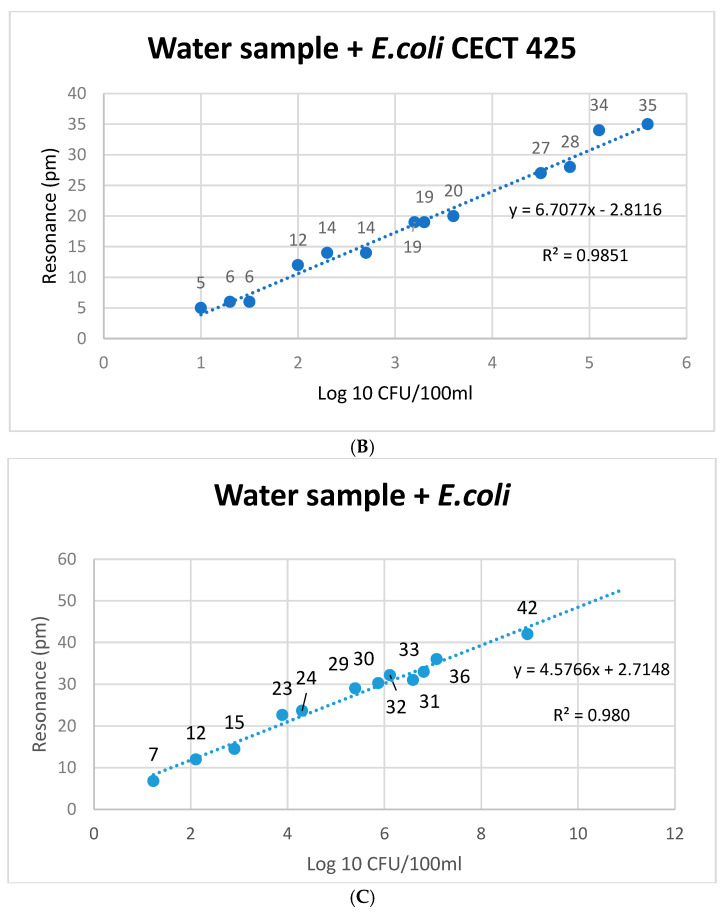
Validation calibration curves for *E. coli* antigens. These curves are divided into three panels. (**A**) Relationship between the resonance shift and the concentration of pure *E. coli* CECT 425 strain in a controlled environment. (**B**) Calibration curve of *E. coli* CECT 425 spiked in drinking water samples. (**C**) Calibration curve of naturally contaminated drinking water samples with *E. coli*. The Limit of Detection (LoD) and Limit of Quantification (LoQ) were computed to specify the minimum concentration of the analyte that the biosensor is capable of reliably detecting and quantifying. The Upper Limit of Quantification (ULOQ) is established in accordance with analysis requirements. In order to ascertain *s*0, no fewer than six determinations of samples at the calculated breakpoint concentration were performed. The concentration data for *E. coli* strains and both spiked and naturally contaminated samples were sourced from Tables S1 and S2 (Supplementary information attached file), with resonance measured in picometers (pm) using a laboratory setup reader, as detailed in Section 2.
