# Supplementary material for: A Photonic Immunosensor Detection Method for Viable and Non-Viable E. coli in Water Samples
Source: Microorganisms. 2024 Jun 29;12(7):1328. doi: 10.3390/microorganisms12071328 (PMC11278787; doi:10.3390/microorganisms12071328)
Supplement: Supplementary file 1 [file microorganisms-12-01328-s001.zip › microorganisms-3059044-supplementary.pdf]

Supplementary information attached file

Table S1. Detection inmunosensor results against *E.coli*. This analysis includes a list of serial samples quantified of the *E.coli* CECT425 strain and E.coli CECT425 spiked on water for human consumption samples without the confirmed presence of *E.coli*. The evaluation aimed to assess the performance of the detection method across different operators and measurement equipment, but on identical samples.

Target sample*	Inmunosensor response**	Quantitative Enumeration (CFU/100 ml)***
E.coli CECT425 01/2023	+	2,5 x 10^6
E.coli CECT425 01/2023	-	0
E.coli CECT425 01/2023	+	2,5 x10^5
E.coli CECT425 01/2023	+	2,5 x 10^4
E.coli CECT425 01/2023	+	4 x 10^3
E.coli CECT425 03/2023	+	4x 10^1
E.coli CECT425 03/2023	+	4 x 10^2
E.coli CECT425 03/2023	+	3,8 x *10^6*
E.coli CECT425 03/2023	+	3,8 x 10^4
E.coli CECT425 03/2023	+	3,8 x 10^5
E.coli CECT425 04/2023	-	0

E.coli CECT425 04/2023	+	*1 x10²*
E.coli CECT425 04/2023	+	1x 10^3
E.coli CECT425 04/2023	-	0
E.coli CECT425 04/2023	-	1x 10^1
Water sample + CECT425	+	2 x 10^3
Water sample + CECT425	+	4,5 x 10^5
Water sample + CECT425	+	2,0 x 10^2
Water sample + CECT425	+	3,2 x 10^1
Water sample + CECT425	+	3,0 x 10^5
Water sample + CECT425	+	4,6 x 10^3
Water sample + CECT425	+	5,2 x 10^2
Water sample + CECT425	+	2,0 x 10^1
Water sample + CECT425	+	1,0 x 10^2
Water sample + CECT425	+	2,0 x 10^1
Water sample + CECT425	+	1,5 x 10^5
Water sample + CECT425	+	6,2 x 10^4
Water sample + CECT425	+	1,5 x 10^3
Water sample + CECT425	+	4,6 x 10^2
Water sample + CECT425	-	0
Water sample + CECT425	+	1,0 x 10^1

* Samples were in chronological order: inoculum strain CECT425 and water for human consumption samples spiked with *E.coli* strain CECT 425.
** + Inmunosensor detection of *E.coli*.- Absence of *E.coli*.
*** *E.coli* artificial contamination was estimated based on the concentration of Colony Forming Units (CFU) per 100 milliliters (CFU/100 mL) in the first dilution of each sample. This measurement reflects the number of *E.coli* entire bacteria in the initial sample.

Table S2. Detection inmunosensor results of water for human consumption samples naturally contaminated by *E.coli*. This analysis includes a list of quantified samples of serial dilutions of real water for human consumption samples employed in a food industry.

Target sample *	Inmunosensor response**	Quantitative Enumeration (CFU/100 ml)***
Sample 1 01/2003	+	6,5 x10^6
Sanple 2 01/2003	+	1,4 x10^6
Sample 3 01/2003	+	1,3 x10^6
Sample 4 01/2003	+	7,5 x 10^5

Sample 5 01/2003	+	2×10^4
Sample 6 03/2003	+	$7{,}8 \times 10^3$
Sample 7 03/2003	-	0
Sample 8 03/2003	-	0,2
Sample 9 01/2003	+	$8{,}0 \times 10^2$
Sample 10 01/2003	+	$1{,}7 \times 10^1$
Sample 11 01/2003	+	$1{,}2 \times 10^7$
Sample 12 01/2003	+	$2{,}5 \times 10^5$
Sample 13 03/2003	+	$1{,}2 \times 10^8$

* Samples were in chronological order: water for human consumption samples naturally contaminated by *E.coli*.
** + Inmunosensor detection of *E.coli* - Inmunosensor Absence response *E.coli*.
*** *E.coli* natural contamination was estimated based on the concentration of Colony Forming Units (CFU) per 100 milliliters (CFU/100 mL) in the first dilution of each sample. This measurement reflects the number of *E.coli* entire bacteria in the initial sample.
